# Rising COVID-19 related acute pulmonary emboli but falling national CTEPH referrals from a large national dataset

**DOI:** 10.1183/23120541.00431-2021

**Published:** 2021-09-02

**Authors:** Joseph Newman, Iryna Boubriak, David Jenkins, Choo Ng, Alessandro Ruggiero, Nicholas Screaton, John Cannon, Mark Toshner

**Affiliations:** 1 Royal Papworth Hospital, Cambridge, UK; 2 Dept of Medicine, Heart Lung Research Institute, University of Cambridge, Cambridge, UK

## Abstract

Chronic thromboembolic pulmonary hypertension (CTEPH) is an uncommon but significant complication of acute pulmonary embolism (PE). There is a strong documented causal relationship between Covid-19 and venous thromboembolism. Despite this, we have observed a seemingly dichotomous reduction in the rate of new CTEPH referrals during the pandemic to our national specialist centre. Royal Papworth is the UK’s only quaternary CTEPH centre and captures all new diagnoses.

This is a PDF-only article. Please click on the PDF link above to read it.

